# Relating Inter-Individual Differences in Verbal Creative Thinking to Cerebral Structures: An Optimal Voxel-Based Morphometry Study

**DOI:** 10.1371/journal.pone.0079272

**Published:** 2013-11-05

**Authors:** Feifei Zhu, Qinglin Zhang, Jiang Qiu

**Affiliations:** 1 Key Laboratory of Cognition and Personality (SWU), Ministry of Education, Chongqing, China; 2 School of Psychology, Southwest University, Chongqing, China; Institute of Psychology, Chinese Academy of Sciences, China

## Abstract

Creativity can be defined the capacity of an individual to produce something original and useful. An important measurable component of creativity is divergent thinking. Despite existing studies on creativity-related cerebral structural basis, no study has used a large sample to investigate the relationship between individual verbal creativity and regional gray matter volumes (GMVs) and white matter volumes (WMVs). In the present work, optimal voxel-based morphometry (VBM) was employed to identify the structure that correlates verbal creativity (measured by the verbal form of Torrance Tests of Creative Thinking) across the brain in young healthy subjects. Verbal creativity was found to be significantly positively correlated with regional GMV in the left inferior frontal gyrus (IFG), which is believed to be responsible for language production and comprehension, new semantic representation, and memory retrieval, and in the right IFG, which may involve inhibitory control and attention switching. A relationship between verbal creativity and regional WMV in the left and right IFG was also observed. Overall, a highly verbal creative individual with superior verbal skills may demonstrate a greater computational efficiency in the brain areas involved in high-level cognitive processes including language production, semantic representation and cognitive control.

## Introduction

Creativity refers to the ability of an individual to produce novel and practical products or points of view [1]. Such ability is known to serves as pillars of society, affecting all aspects of human life [2,3]. The progress and innovation of human society rely on the ability of individuals to step out of the box and breathe new life to things [3]. Hence, creativity is essential to the development of human civilization and plays a crucial role in cultural life. Modern creativity research is attributed mainly to Joy Paul Guilford in 1950. Guilford indicated that creative thinking is the concrete manifestation of individual creativity, and that divergent and convergent thinking together constitute complete creative thinking, the core of which is divergent thinking [4]. Divergent thinking refers to the ability of an individual to develop several solutions to a highly complex open-ended problem [5]. This component of creative thinking has attracted considerable attention because it characterizes a technique for pushing difficult problems into the realm of empirical science [3]. This focus on divergent thinking has led to the development of several standardized psychometric instruments of creativity. The Torrance Test of Creative Thinking (TTCT) [6] is a well-established creativity test for measuring creative thinking focusing on divergent thinking. Many experimental paradigms, developed from TTCT, have been applied in laboratory-based research on creativity. With the emergence of cognitive neuroscience, new research techniques, including high temporal resolution brain electrophysiology such as electroencephalograph (EEG), event-related potential (ERP) and high spatial resolution neuroimage techniques such as diffusion tensor imaging (DTI) and functional magnetic resonance imaging (fMRI) have been applied in cognitive psychology. These approaches, along with behavioral assessment tools on creativity, can help researchers explore the neural mechanism of the creative thinking process.

Efforts have focused on creativity-related functional brain areas [7-15]. In their review, Fink and Benedek (2012) argued that in focusing on one measure of brain activation (i.e., alpha power in the EEG), task- or event-related increases in alpha synchronization during creative ideation were often seen at the prefrontal and parietal and occipital sites [16,17]. For instance, Fink and Neubauer (2006) conducted an EEG study on creativity, and aimed to explore the following two tasks derived from TTCT: a) an unusual situation in that requires explanation, such as “a light in the darkness”; and b) a utopian situation that will never actually happen, such as “Imagine, there were a creeping plant rising up to the sky. What would you await at the end of this plant?” The participants of both tasks were asked to imagine themselves in the given situations and to visualize possible causes and outcomes. The result of the study reflected a frontal alpha synchronization during the performance of the tasks, indicating that the generation of novel ideas involves an top-down inhibition function in the prefrontal cortex and a right parietal alpha synchronization involved in vivid imaginative abilities during creative ideation [7]. In another review, Dietrich and Kanso (2010) suggested that in focusing on the functional aspects of brain activity, such as fMRI, PET, and NIRS, and on the well-established creative thinking process (or more generally on divergent thinking), difficulties in identifying replicable brain correlates underlying creativity were apparent [3]. Despite the diversity of the functional activation patterns among these neuroimaging studies, the activation of prefrontal regions was consistently reported [3]. The authors argued that the data permit the conclusion that the prefrontal cortex has a key role in divergent thinking. Beyond this rather general statement, however, a more specific premise is not possible [3].

Aside from the task-related functional research on creativity mentioned above, a few recent studies have focused on the cerebral structural basis of creativity and on the inter-individual differences in creativity [14,16,18-22]. Overall, similar to the diversity of the creativity-related functional brain areas, the creativity-related brain structures were also scattered and diffused in different studies. For example, Jung et al. (2010) observed a relationship between creativity and cortical thickness in several brain regions including the lingual gyrus, the right posterior cingulate, the left lateral orbitofrontal and the right angular gyrus [14]. In terms of white matter microstructure, creative performance was found to be positively correlated with white matter integrity, as assessed by fractional anisotropy (FA)，in the left superior frontal gyrus [18]. Takeuchi et al. (2010a) reported a positive correlations between regional GMV and individual creativity in several regions known to be associated with the dopaminergic system, such as the right dorsolateral prefrontal cortex (DLPFC), the bilateral striata, the substantia nigra, tegmental ventral area and periaqueductal [20]. Another study conducted by Takeuchi et al. (2010b) suggested that individual creativity is remarkably positively correlated with FA in the bilateral prefrontal cortices, the body of the corpus callosum, the bilateral basal ganglia, the bilateral temporo-parietal junction and in the right inferior parietal lobule [19]. Fink et al. (2013) reported that verbal creativity was significantly and positively associated with gray matter density (GMD) in clusters within the right cuneus and the right precuneus [16]. The inconsistency of these studies may be primarily due to the broad diversity in measuring creativity as well as to the diversity of experimental procedures and methodologies used in this field of research. For instance, the discrepancies between Takeuchi et al. (2010a) and Jung et al. (2010) might be due to the neuroimaging method used (GMV with VBM in Takeuchi et al. 2010a vs. cortical thickness in Jung et al2010), but also to the psychometric measures that they used to assess ‘‘creativity’’ (verbal divergent thinking tasks were used to measure creativity in Takeuchi et al. 2010a vs. both verbal and figural divergent thinking tasks in Jung et al. 2010). The discrepancies between Takeuchi et al. (2010a) and Fink et al. (2013) might be due to the difference in the index of brain structures that were measured [GMV in Takeuchi et al. (2010a) vs. GMD in Fink et al. (2013)]. In addition, in closely examining these results, we could see that all these studies, except Fink et al. (2013), reported that prefrontal brain areas correlated with creativity. 

Creativity is not a prime example for a unitary or homogeneous construct [3,17]; hence, neuroscientists have faced many challenges to decompose the multifaceted construct of creativity into smaller and more definable units (i.e., verbal creativity, figural creativity, and art creativity), and to specifically relate these subcomponents of creativity to both the structural and functional parameters of the brain. Several studies challenged this issue [e.g., Takeuchi et al. (2010a) and Fink et al. (2013)]. In addition, one study [22], aimed at examining the relationships between brain connectivity and visuospatial divergent thinking (measured by the figural TTCT) chose the corpus callosum as a region of interest (ROI) because of the postulate that inter-hemispheric connectivity is critical for divergent thinking. The authors found that the creative visuospatial performance is inversely related to the size of the corpus callosum. They suggested that decreased callosal connectivity enhances hemispheric specialization. In particular, the callosal sectioning prevented the visuospatial analysis performed by the right hemisphere from reaching the verbal left hemisphere [22]. As a supplement for the neural anatomy of creative visuospatial performance, Gansler et al. (2011) applied a statistics analysis of ROIs to explore the gray matter structure associated with creative visuospatial performance (also assessed by the figural TTCT) the authors found that creative visuospatial performance is associated with GMV in the right parietal lobe, which is believed to be important in visuospatial processing [21]. The study further reported that visuospatial divergent thinking requires some specialized cerebral functions (visuospatial imagery and skills) that may be mediated by the right parietal lobe.

In this study, we focused on verbal creativity measured by the verbal TTCT and on the neural correlates of verbal creativity to complement the structural brain research on definable units of creativity. The verbal TTCT comprises seven verbal tasks or items. The tasks applied in Takeuchi et al. (2010a) and Fink et al. (2013) are only a part [three tasks in Takeuchi et al. (2010a) and one task in Fink et al. (2013)] developed from the verbal TTCT. We thought that using all seven tasks from the verbal TTCT might provide a higher validity because in the same reliability, validity increases with the increase in the items. In terms of the index of brain structures, GMV, rather than GMD, was obtained with an optimal voxel-based morphometry (VBM), which is an automated technique for assessing structural changes in the brain. (see Images preprocessing for VBM, Method in details). One purpose of our study was to verify whether these brain correlates underlying verbal creative thinking in Takeuchi et al. (2010a) and Fink et al. (2013) can be stably manifested in our replicable experiment. Conversely, we wondered that if healthy participants with greater visuospatial creativity have larger GMVs in the right parietal lobe, then people with different verbal creative ability may also demonstrate differences in the cortical regions involving language cognition and divergent thinking. Specifically, the bilateral prefrontal cortices might be the cortical areas where these differences may likely be manifested. As mentioned above, the prefrontal cortex is known to play an important role in creative thinking. A recent study associated the left inferior frontal gyrus (IFG) activation with novelty-based representations originating from the development and selection of semantic relatedness [23]. In addition, language production and comprehension are known to be mediated by Broca’s area (BA44 and BA45), which is a part of the left IFG [24-26]. Based on the idea that the volume of cortical tissue devoted to a certain function influences the quality of a person’s ability to perform that function, verbal creative performance was proposed to be associated with cerebral (GMVs) or/and white matter volumes (WMVs) in the brain regions that are believed to be important in verbal processing and divergent thinking, such as Broca’s area or the left IFG and bilateral prefrontal cortices.

## Methods

### Participants

A total of 285 subjects (130 men, aged 17-26 years, mean =20.18 ± 1.27 years; 155 women, aged 17-24 years, mean = 19.68 ± 1.01 years) participated in the study as part of our ongoing project investigating the associations among brain imaging, cognition, emotion and personality. The data collected from the participants in this work will be used in other studies exploring a different subject matter. All participants were right-handed, had normal or corrected-to-normal vision, and had no history of neurological or psychiatric illness. All participants recruited are undergraduates or graduates in Southwest University. They gave their informed written consent and we also obtained informed written consent from the two youngest participants’ (aged 17 years old) guardians who were their college instructors. The local ethics committee of Southwest University (Chongqing, China) approved this consent procedure and the experiment procedure which were in accordance with the standards of the Declaration of Helsinki. 

### Assessment of creativity

The TTCT was designed as a measure of divergent thinking, which is a central aspect of creativity [27]. The TTCT contains verbal, figural and auditory tests [27]. In this study, the verbal TTCT was used to assess individual divergent thinking abilities [28-30]. The verbal TTCT comprises seven tasks. Three of the tasks required participants to generate questions, causes and consequences in response to a scenario presented pictorially (given 5 minutes, respectively). The fourth task required participants to propose creative ideas to improve a toy elephant (e.g., remake this toy elephant as a money-box or pillow; given 10 minutes). The fifth task asked participants to generate different ideas for the use of cardboard boxes (e.g., the cardboard boxes can be used as feeder or bathtub for animals; given 10 minutes). The sixth task required participants to think of questions relating to a carton (e.g., why people make carton; given 5 minutes). Finally, the seventh task asked participants to imagine the consequences of an imaginary scenario (given 5 minutes) [31]. Scoring comprised three components: fluency (the number of meaningful and relevant responses, which is associated with the ability to generate and consider other possibilities), flexibility (the number of different categories of responses, which reflects the ability to shift between conceptual fields) and originality (the degree of originality of the responses, which is associated with thinking “outside the box”) [31]. The participates’ responses were evaluated according to the norming scoring guides [32-34]. More specifically, the fluency represents the number of meaningful responses, the flexibility represents how many different categories to which the responses are belong (e.g., the cardboard boxes can be used as shoes or clothes which are belong to dress category, therefore, the two responses only scoring 1 point), and the originality represents the degree of originality of the responses (e.g., the cardboard boxes can be used as postcard scoring 2 point, but the response the cardboard boxes can be used as vase scoring 0 point). Three raters took part in the evaluation; the inter-rater correlation coefficient was 0.9. In the current study, the total scores (sum of fluency, flexibility, and originality scores) were used as the creativity index. Heausler and Thompson (1988) revealed that the total score was highly correlated with the scores of the three components (fluency, flexibility, and originality), and the scores of the three components were highly correlated with each other (all correlations between the scores had simple correlation coefficients of >0.81). Heausler and Thompson (1988) suggested that the high correlations among the three subscales of the TTCT could not provide meaningfully different data, and therefore, the total score of TTCT can be used as valid creativity index [35].

### Assessment of general intelligence

In order to examine intellectual ability, participants completed the Combined Raven’s Test (CRT)-the Chinese revised edition. The reliability coefficient was 0.92 [36,37]. The CRT included the Raven's standard progressive matrix (C, D, E sets) and Raven's colored progressive matrix (A, B, AB sets), consisted of 72 items revised by the Psychology Department of East China Normal University in 1989. The score of this test (the number of correct answers given in 40 minutes) was used as a psychometric index of individual intelligence. In line with standard practice, the current study focused on the total score of the test [38] [39].

### Images acquisition

All images were collected using a 3-T Siemens Trio MRI scanner (Siemens Medical, Erlangen, Germany). The high-resolution T1-weighted structural images were acquired using a magnetization-prepared rapid gradient echo (MPRAGE) sequence. The parameters were as follows: TR = 1900ms, TE = 2.52 ms, TI = 900 ms, FA = 9 degrees, 256 × 256 matrix, 176 slices, 1.0 mm slice thickness, voxel size = 1×1×1 mm.

### Images preprocessing for VBM

All images were processed using the SPM8 (Wellcome Department of Cognitive Neurology, London, UK; www.fil.ion.ucl.ac/spm) implemented in Matlab 7.8 (MathWorks Inc., Natick, MA, USA). First, each MR image was displayed in SPM8 to monitor artifacts or obvious anatomical abnormalities. For enhanced registration, the reorientation of the images was manually set to the anterior commissure. Then, VBM with diffeomorphic anatomical registration was performed using exponentiated lie algebra (DARTEL) [40]. DARTEL is proven as be an optimal VBM procedure that generates a more precise registration than the standard VBM procedure [41]. The New Segment Toolbox from SPM8 was applied to every T1-weighted MR image to extract tissue maps corresponding to gray matter, white matter, and cerebral spinal fluid in native space. This algorithm is an improved version of the unified segmentation algorithm [42]. It uses a Bayesian framework to respectively carry out the probabilistic tissue classification and spatial non-linear deformation to Montreal Neurological Institute (MNI) space. The segmented images of gray and white matter were aligned and warped to a template space. The images were then resampled to 1.5 mm isotropic voxels. Using the DARTEL template-creation toolbox, the resliced images of gray and white matter were then registered to a subject-specific template to improve inter-subject alignment. Subsequently, the normalization function in the DARTEL toolbox was used to normalize the individual images of gray and white matter to MNI space (1.5 mm isotropic voxel). Finally, the gray and white matter map of each subject were warped using their corresponding smooth, reversible deformation parameters to the custom template space and then to the MNI standard space. As for GMV and WMV, the warped images of gray and white matter were modulated by calculating the Jacobian determinants derived from the special normalization step and by multiplying each voxel by the relative change in volume [43]. The modulation step was carried out to correct any volume changes during nonlinear normalization. The warped modulated images of gray and white matter were smoothened through the convolution of a 10-mm full-width at half-maximum isotropic Gaussian kernel.

### Statistical analysis

In the whole brain analysis, a multiple regression model in SPM8 was applied to determine the GMV or WMV that displayed considerable covariation with verbal creativity measured by TTCT respectively. In terms of GMV, to deal with individual differences in brain size, the value of total gray matter volume, obtained by extracting values in the non-normalized segmented images of gray matter, was used to adjust for individual differences in brain size. In terms of WMV, the value of total white matter volume, obtained by extracting values in the non-normalized segmented images of white matter, was used to adjust for individual differences in brain size. For each regression model, repressors of gender, age, general intelligence, and total gray matter volume or total white matter volume were incorporated into the design matrix as covariates of no interest. Any effects correlated with the aforementioned factors were thus regressed. Explicit masking was also applied with population-specific optimal threshold achieved by the masking toolbox in SPM8 to restrict the search volume within gray and white matter (http://www0.cs.ucl.ac.uk/staff/g.ridgway/masking/). Contrary to absolute or relative threshold masking, explicit masking reduced the risk of false negatives caused by overly restrictive masking, as potentially interesting voxels are excluded from the statistical analysis [44]. The T-statistic maps that show the correlation between creativity and regional GMV and WMV were reported. In addition, the voxels that survived a cluster-level statistical threshold (corrected for non-stationary, p < 0.05) with an underlying uncorrected voxel level of p < 0.0001 were identified. Non-stationary cluster-size correction can be applied to data known to be non-stationary data (in another words, not uniformly smooth data), such as VBM data [45]. In this non-stationary cluster-size correction of random field theory, a lower cluster determining threshold resulted in a more conservative test [45].

Next, interest in sex effect, whether interaction effects existed between sex and verbal creativity on GMV, was investigated. In the whole brain analysis, a voxel-wise analysis of covariance (ANCOVA) was used, and added sex difference as a group factor using the full factorial option of SPM8 [46]. Age, general intelligence, the total scores of TTCT, and total gray matter volume or total white matter volume were entered as covariates of no interest. The interaction effect between sex and the total score of TTCT on GMV was assessed using t-contrasts. The voxels that survived a cluster-level statistical threshold (corrected for non-stationary, *p* < 0.05) with an underlying uncorrected voxel level of *p* < 0.0001 were identified.

## Results

### Behavioral Data

The intelligence measured by CRT was not significantly correlated with age, the three sub-dimensional scores of verbal TTCT (originality, flexibility, and fluency), and with the total score of verbal TTCT. Meanwhile, the correlations among originality, flexibility, fluency, and the total score of verbal TTCT were high (r > 0.57, p = 0.000), which is consisting with findings of Heausler and Thompson [35]. The distribution of the total score of verbal TTCT is close to normal distribution (Skewness = 0.53, Std. Error of Skewness = 0.14; Kurtosis = -0.08, Std. Error of Kurtosis = 0.29). Extreme values were detected by the criterion, which is range within Mean plus or minus Standard deviation multiplied by three. We initially calculated the Mean and Standard deviation of the total scores of TTCT, then obtained the upper limit by obtaining Mean plus Standard deviation multiplied by three, and the lower limit by obtaining Mean minus Standard deviation multiplied by three. We deleted the values greater than the upper limit and less than the lower limit. No extreme values in the measures on verbal creativity were derived. The same criterion was used to detect extreme values on general intelligence. Two extreme values less than the lower limit (the score <56) were derived; thus, we deleted the two participants’ data corresponding to the two extreme values. Finally, data on 283 participants were retained to perform subsequent analysis. Table 1 shows the descriptive statistics of the 283 study participants.

**Table 1:** Descriptive statistics of behavioral measures.

	males		females	
	means	SD	means	SD
Age	20.17	1.28	19.68	1.01
CRT	65.69	3.53	66.25	3.06
Originality	43.47	14.52	47.61	13.16
Flexibility	28.59	5.55	27.54	5.25
Fluency	54.8	16.22	57.61	14.42
TTCT	123.59	33.89	132.39	30.4

### Correlation between GMVs and verbal creativity

**Figure 1:**
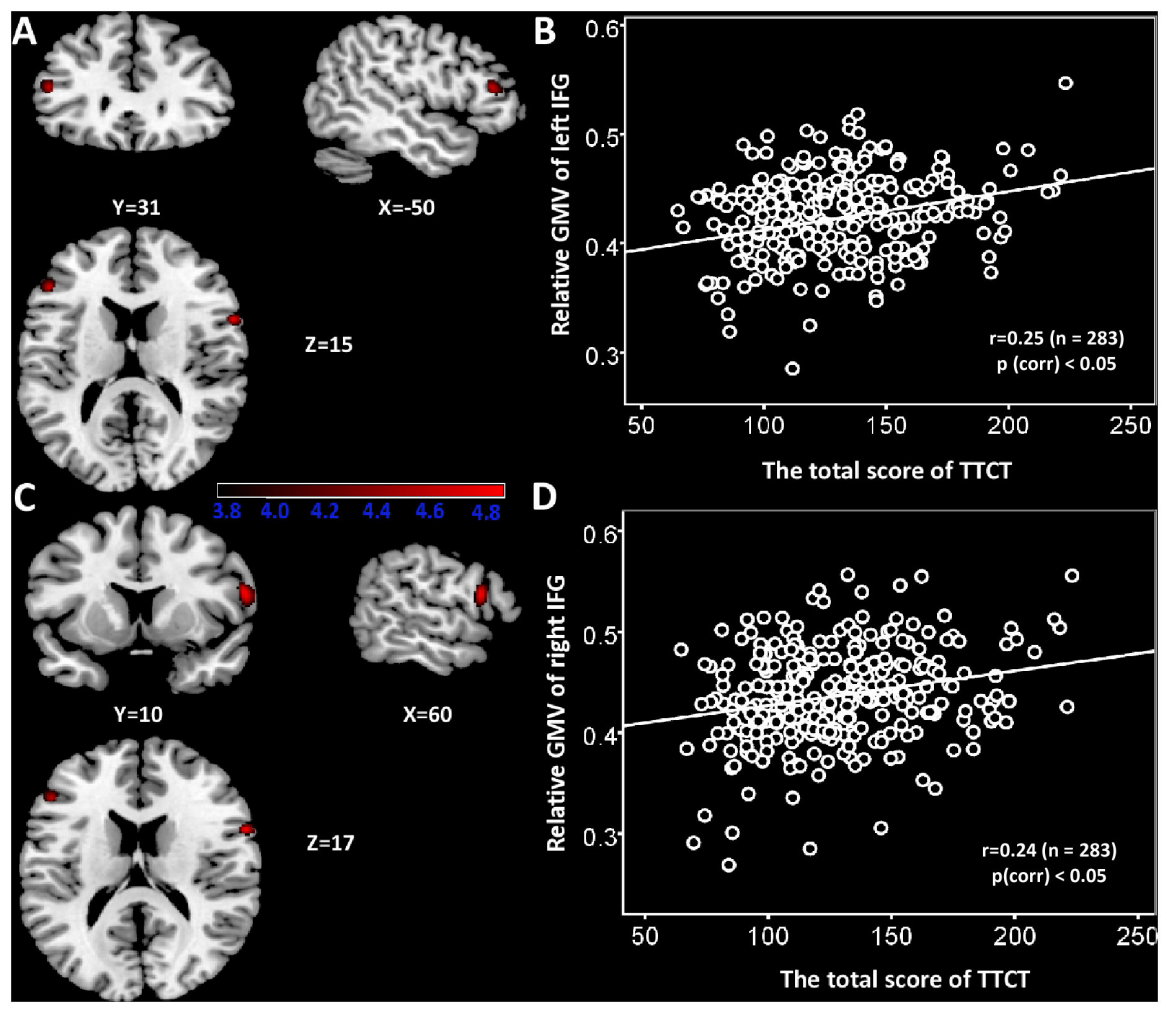
The relationship between regional GMV and verbal creativity. (A) Statistical parametric mapping for the covariation between subjects’ verbal TTCT total score and regional GMV, the left IFG (BA45) is significantly positive associated with verbal TTCT total sore. The significant cluster is p < 0.05 nonstationary corrected with an underlying uncorrelated voxel level at p<0.0001. Structural maps shown at sagittal, coronal and axial sections are overlaid on the T1-weighted images. The significant cluster is shown at t > 3.8 for visualization purpose. (B) A scatterplot between verbal TTCT total score and relative GMV of the left IFG adjusted for age, gender, and general intelligence is shown for illustration purpose only. (C) The right IFG in which variability in regional GMV exhibited significant positive correlation with verbal TTCT total score is superimposed on a standard T1-weighted template brain in MNI stereotactic space. (D) A scatterplot between verbal TTCT total score and relative GMV of right IFG adjusted for age, gender, and general intelligence is shown for illustration purpose only.

After control for age, gender, general intelligence, and total gray matter volume in the multiple regression analysis, the correlation of GMVs with verbal creative thinking measured by verbal TTCT was determined (Table 2). As shown in Table 2, significantly positive correlation was found between the verbal TTCT score and the left IFG, pars triangularis (BA45: x = -50, y = 31, z = 15; see also Figure 1A), and the right IFG (x =60, y = 10, z = 17; see also Figure 1C). All voxels survived at cluster-level non-stationary correction for multiple comparisons (p < 0.05). The average local GMVs (left IFG/BA 45, right IFG) were then obtained using the “Extract ROIs signal” utilities implemented in REST [47]. To control for individual differences in whole brain size, the average relative GMVs obtained by dividing local GMVs by total gray matter volume was calculated. These average relative GMVs were then used as the dependent variables for all bivariate correlations (Figures 1B and 1D). 

**Table 2:** Regional GMV correlated with verbal creativity.

	**Area**	**T score**	**MNI coordinate**			**Clusters size**
			X	Y	Z	
Positive correlation	Left IFG/BA 45	4.78	-50	31	15	266
	Right IFG	4.82	60	10	17	260
Negative correlation	No					

(voxels survived at clusters-level p<0.05，corrected with non-stationary)

### Correlation between WMVs and verbal creativity

**Figure 2:**
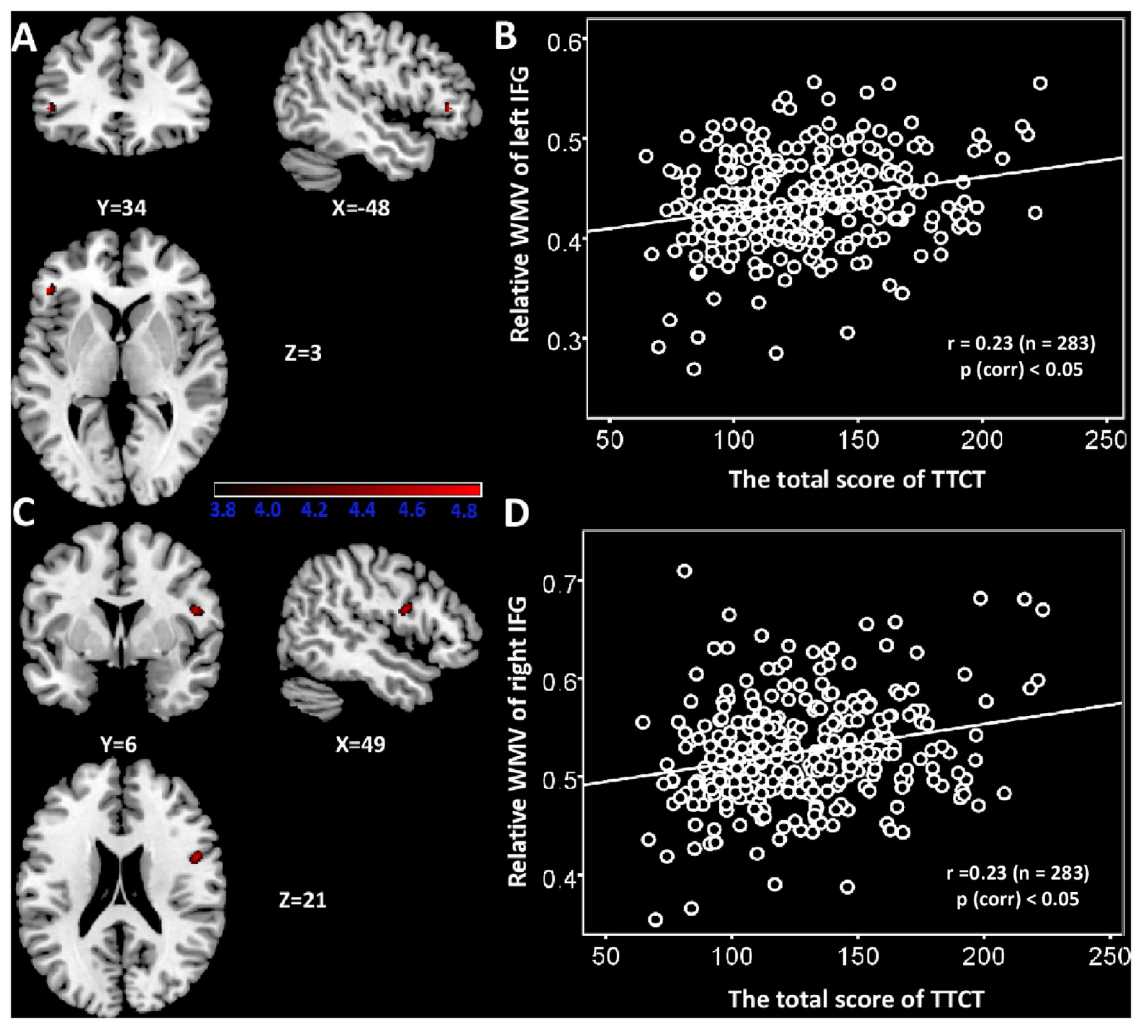
The relationship between regional WMV and verbal creativity. (A) Statistical parametric mapping for the covariation between subjects’ verbal TTCT total score and regional WMV, the left IFG is significantly positive associated with verbal TTCT total sore. The significant cluster is p <0.05 nonstationary corrected with an underlying uncorrelated voxel level at p<0.0001. Structural maps shown at sagittal, coronal and axial sections are overlaid on the T1-weighted images. The significant cluster is shown at t > 3.8 for visualization purpose. (B) A scatterplot between verbal TTCT total score and relative WMV of the left IFG adjusted for age, gender, and general intelligence is shown for illustration purpose only. (C) The right IFG in which variability in regional WMV exhibited significant positive correlation with verbal TTCT total score is superimposed on a standard T1-weighted template brain in MNI stereotactic space. (D) A scatterplot between verbal TTCT total score and relative WMV of the right IFG adjusted for age, gender, and general intelligence is shown for illustration purpose only.

Table 3 shows the correlation of WMVs with verbal TTCT. The regional WMVs of left IFG (x = -48, y = 34, z = 3; see also Figure 2A) and right IFG (x = 49, y = 6, z = 21; see also Figure 2C) were found to be significantly positively correlated with the total score of verbal TTCT. No negative correlation was found between regional WMV and the total score of verbal TTCT after multiple comparisons. All voxels survived at cluster-level non-stationary correction for multiple comparisons (p < 0.05). Figure 2 shows the bivariate correlations between relative WMVs in the left/right IFG and the verbal TTCT score. Relative WMVs were obtained using the same approach as that used for relative GMVs.

**Table 3:** Correlation between WMV and TTCT total score.

	**Area**	**T score**	**MNI coordinate**			**Clusters size**
			X	Y	Z	
Positive correlation	Left IFG	4.50	-48	34	3	21
	Right IFG	4.43	49	6	21	44
Negative correlation	No					

(voxels survived at clusters-level p<0.05 corrected with non-stationary)

### Effects of Interaction between Sex and Verbal Creativity on the Regional GMVs and WMVs

The ANCOVA using data from both sexes revealed no significant effects of the interaction between the total scores of TTCT and sex on the regional GMVs and WMVs.

## Discussion

To address the association of cortical specialization to verbal creativity, the present study investigated the neural correlates of performance on verbal creativity tasks. The results of multiple linear regressions showed that increased regional GMV in the left IFG (BA45) and the right IFG is associated with verbal creativity measured by the verbal form of TTCT. Consistent with the assumption made in this study, the analysis demonstrated specificity to the prefrontal cortex. Existing evidences have also proven that prefrontal cortices are responsible for tasks requiring divergent thinking [9,20]. Furthermore, verbal creative performance was found to be positively correlated with regional WMVs in the left and right IFG as well. 

With regard to gray matter structures, Takeuchi et al. (2010a) reported that individual differences in creativity (actually verbal creativity) were associated with regional GMV in the DLPFC and several cortical-subcortical regions. Fink et al. (2013) also reported that verbal creativity was significantly and positively associated with GMD in clusters within the right cuneus and the right precuneus. The lack of a relationship between verbal creativity and the prefrontal cortex is a slightly surprising finding in the work of Fink et al. (2013). The discrepancies in these results might be attributed to first, the methodological difference in the experiments. As the authors [Takeuchi et al. (2010a)] pointed out, the Standard VBM procedure applied in their experiment [in Fink et al. (2013) as well] had been criticized because of a few methodological limitations, such as the local misregistration of images in spatial normalization and a simple linear relationship assumed in segmentation [20]. This condition might result in several “pseudo-brain-correlates” that could not be replicated in a similar research. VBM with diffeomorphic anatomical registration was performed using exponentiated lie algebra (DARTEL) in our study. As described in the Method, DARTEL is proven as be an optimal VBM procedure that generates a more precise registration than the standard VBM procedure [38]. Meanwhile, a Bayesian framework was used to respectively carry out the probabilistic tissue classification (segmentation) and spatial non-linear deformation to Montreal Neurological Institute (MNI) space. In other words, the methodological limitations mentioned above were improved in our study. The brain correlates underlying verbal creativity—the subcortical regions in Takeuchi et al. (2010a) and the right cuneus and the right precuneus in Fink et al. (2013)—primarily might be the “pseudo-brain-correlates.” Second, the discrepancies in the results might be attributed to the different index measuring gray matter structures. We initially need to comprehend the different meanings of GMD and GMV. GMD represents the relative concentration of gray matter structures in spatially warped images (i.e., the proportion of gray matter relative to all tissue types within a region), whereas GMV represents the absolute amount of gray matter [48]. Several studies have indicated that the increase in GMV may be attributed to synaptogenesis, whereas the increase in WMV may be attributed to intracranial myelination [49,50]. Therefore, in regions where GMV increases but where GMD does not significantly change, the increase in GMV may be attributed to an increase in the number of synapses per neuron; a similar increase in the WMV due to myelination occurs simultaneously in these regions [48]. Both GMVs and WMVs in the bilateral prefrontal regions apparently increased with verbal creativity in the present study, whereas GMD in the regions did not significantly change with verbal creativity in Fink et al. (2013).

With regard to the white matter structures, the inconsistencies with previous studies were also observed. The paradoxical results of white structure related to creativity might be attributed to first, the difference in the experimental tasks. In Jung et al. (2010), both verbal and visuospatial divergent-thinking capacities were assessed, whereas in the present study and in Takeuchi et al. (2010b), only the verbal divergent-thinking capacity was assessed. Second, the paradoxical results might be attributed to the methodological difference in the experiments [FA derived from DTI in Jung et al. (2010) and Takeuchi et al. (2010b) vs. WMV derived from VBM in the present study]. A direct link between macroscopic neural structure (WMV) and microscopic neural structure (FA) in the human brain has yet to be established. What is known is that white matter structures are largely composed of nerve fibers called axons and fatty myelin sheaths around the axons [51]. As mentioned above, the increase in WMV may be attributed to intracranial myelination [46,47]. The myelination process allows neural signals to propagate more swiftly and with less signal loss. This aspect enhances connectivity within specific brain regions (the bilateral IFG here) and improves broader neural pathways connecting spatially separate regions required for many sensory, cognitive, and motor functions. The neural pathways are extremely complicated and require further research. Overall, the greater GMVs, together with the greater WMVs in the bilateral IFG, may reveal a greater neural computational efficiency in the regions.

The bilateral IFG (including BA 45) was the only region found to be associated with verbal creative thinking in the present study. To our knowledge, BA45, together with BA44, comprises Broca’s area, a region that is mainly involved in language production and comprehension, such as semantic generation tasks [52] and verbal fluency [53]. In one study, neuropsychological evidence revealed that patients with left prefrontal lesions exhibited impairment of the generation of verbal fluency [54]. Moreover, the role of the left inferior prefrontal cortex in ideas generation has been supported by a considerable amount of neuroimaging data [55,56]. In the present study, regional GMV in BA45 exhibited a positive correlation with verbal creativity. In other words, individuals with larger GMV in BA45 exhibited higher verbal creative than those with smaller GMV. Larger cortical volume is often linked to the improved computational efficacy of such region [57]. That is, the generation of verbal creative ideas may require a cerebral structural basis that can support effective semantic or concept generation. This view is consistent with the idea that creative processing requires cognitive control over conceptual knowledge networks as that are facilitated by the left ventrolateral prefrontal cortices [58]. In addition, the triangularis part of the left IFG has also been shown to play a role in the cognitive control of memory [59,60]. According to the “two-part” model of memory retrieval [60], we try to retrieve information in a top-down manner when we confront problems or tasks that must be solved. The retrieved knowledge is initially stored in the lateral temporal cortex, such as in the middle temporal gyrus. Recalling the top-down retrieval depends on conscious cognitive control. One can assume that a technique may be employed to exclude irrelevant data from the retrieval knowledge. To hone in on the desired information, some selection must occur. This selection is thought to occur post-retrieval in the left mid-ventral lateral prefrontal cortex (VLPFC), which corresponds to the location of the left IFG, specifically the pars triangularis [60]. Apparently, the process of excluding irrelevant information in top-down memory retrieval plays a crucial role in creative problems solving. The creative process entails activating remote conceptual networks selectively and inhibiting related semantic information [61]. For instance, a previous fMRI study suggested that the left IFG is recruited in the selection of more semantically distant demand [62]. The finding is in line with the idea that the generation of creative ideas involves remote conceptual association [23]. In another fMRI study (Fink et al., 2009), Alternative Use Task and name invention task asking for generating as original names as possible were applied. The two tasks were found to elicit consistently strong activation in the left IFG [9]. The authors suggested that the left IFG is responsible for the emergence of a new semantic representation in divergent thinking. Based on the findings of previous studies, the left IFG is believed to play a major role in the generation of semantic relatedness and in the selection of semantic relatedness. Generally, researchers believe in the existence of structural bases that underlies specific cognitive function. Hence, highly creative individual in the present study had larger GMV in the left IFG (i.e., pars triangularis).

The right IFG is commonly known to play a role in response inhibition [63-65]. Specifically, the right IFG is involved in inhibitory control over cognitive mental processing rather than in motor response control [65]. In addtion, strong evidence suggests that the right IFG also plays a role in attention switching [66,67]. As noted by Hampshire et al. (2010), the right IFG facilitated attention switching by inhibiting the previously attended object, location, or dimension. In this case, the attention shifts. To overcome the pre-potent response, a greater degree of inhibition is therefore necessary when switching attention away from a previous routine response, for example, during the generation of creative ideas. Flexible cognitive control, which is in the form of resistance from distraction (inhibitory control) and anttentional switch may be clearly necessary to sustain creative performance [68]. A recent patient study investigated the association between lesion locations and original responses using creative thinking tasks. Lesions in the right medial prefrontal cortex was found to be correlated with impaired originality [69]. This finding supports that of the current study, wherein highly creative individuals demonstrated larger GMV in the right IFG. In other words, the recruitment of cognitive control is beneficial to the creative process.

At last, the data of the present study did not reveal a dominant cerebral hemisphere in creative processing, as our present data shown. Dietrich and Kanso (2010) reviewed a total of 63 literatures and explored the neural underpinnings of creative behavior using either electroencephalographic techniques or neuroimaging techniques. Notably, these studies did not associated creativity with the right hemisphere or with any part of the right hemisphere, because creative processing does not rely on only a single, simple mental process or brain region. The present study supported this conclusion from a brain structural perspective. Regardless of the indicators of brain structure, i.e. GMVs or WMVs, both the left and right brain regions exhibited an obvious association with creativity.
